# Identification of circRNA-associated ceRNA networks in the longissimus dorsi of yak under different feeding systems

**DOI:** 10.1186/s12917-024-03926-y

**Published:** 2024-02-24

**Authors:** Xiaoming Ma, Xian Guo, La Yongfu, Tong Wang, Pengjia Bao, Min Chu, Xiaoyun Wu, Ping Yan, Chunnian Liang

**Affiliations:** 1 Animal Science Department, Lanzhou Institute of Husbandry and Pharmaceutical Sciences, Chinese Academy of Agricultural Sciences, Lanzhou, China; 2 Key Laboratory of Animal Genetics and Breeding on Tibetan Plateau, Ministry of Agriculture and Rural Affairs, Chinese Academy of Agricultural Sciences, Lanzhou, China; 3 Key Laboratory for Yak Genetics, Breeding, and Reproduction Engineering of Gansu Province, Chinese Academy of Agricultural Sciences, Lanzhou, China; 4 Institute of Western Agriculture, the Chinese Academy of Agricultural sciences, Changji, China

**Keywords:** Yak, Longissimus dorsi, circRNAs, ceRNA

## Abstract

**Background:**

Yaks (Bos grunniens), prized for their ability to thrive in high-altitude environments, are indispensable livestock in the plateau region. Modifying their feeding systems holds significant promise for improving their growth and meat quality. Tenderness, a key determinant of yak meat quality and consumer appeal, is demonstrably influenced by dietary regimen. Indoor feeding regimes have been shown to enhance tenderness by lowering shear stress and optimizing pH values. CircRNAs, well-known modulators of circulatory function, also play a crucial role in skeletal muscle development across various animal species. However, their functional significance in yak skeletal muscle remains largely unexplored.

**Results:**

In this study, we identified a total of 5,534 circRNAs within the longissimus dorsi muscle, and we found 51 differentially expressed circRNAs (20 up-regulated and 31 down-regulated) between the two feeding groups. Constructing a comprehensive ceRNA network illuminated intricate regulatory mechanisms, with *PGP* and circRNA_0617 converging on bta-miR-2285q, mirrored by *KLF15*/circRNA_0345/bta-miR-20b and *CTSF*/circRNA_0348/bta-miR-146a. These findings shed light on the potential of circRNAs to influence yak muscle development and meat quality, offering valuable insights for future research.

**Conclusions:**

This investigation unraveled a complex interaction network between circRNAs、mRNAs and miRNAs in yak skeletal muscle. We further elucidated the target genes regulated by these target genes within the network, offering valuable insights into the potential regulatory mechanisms governing muscle development and meat quality-related traits in yaks.

**Supplementary Information:**

The online version contains supplementary material available at 10.1186/s12917-024-03926-y.

## Background

The yak (*Bos grunniens*) stands as a cornerstone of high-altitude pastoralism in the Qinghai-Tibetan Plateau. Perfectly adapted to this harsh environment, it has thrived for centuries where other livestock wither [1]. Yet, despite their efficient roughage digestion, yak survival and productivity are hampered by the region’s long, cold winters and short growing seasons, leading to meager forage availabilityDespite their efficient digestion of roughage, their survival and productivity are constrained by the scarce forage during the long, cold winters and short growing seasons of the region [2]. Supplementary feeding, particularly during winter, becomes essential for ensuring adequate growth, reproduction, and survival. This practice not only safeguards the animals but also protects the delicate ecological balance of the plateau by mitigating overgrazing pressures [3].

Skeletal muscle is a cornerstone of mammalian movement and energy metabolism. Surrounding tissues, through metabolic processes like glucose and fatty acid oxidation, provide fuel to vital organs [4]. Muscle itself performs the vital conversion of chemical energy into mechanical energy, powering contraction and function. Within muscle, carbohydrates are fully oxidized to CO2 and H2O for energy, a process requiring oxygen. This energy capture often involves the addition of a phosphate group to a molecule with two existing phosphate groups bound to another compound [5]. Glycogen, the primary muscle carbohydrate storage (0.5-1.3% of muscle weight), occurs as solitary particles within the sarcoplasm, nestled between myofibrils and the cell membrane. Enzymes like phosphorylase, pyruvate kinase, and phosphofructokinase-1 regulate glycogen breakdown in muscle cells. While prior studies have linked muscle energy, feeding systems, and meat quality [6, 7], our understanding of muscle development in response to feeding system changes in yaks remains limited. A comprehensive multi-omics approach is vital to elucidate muscle development mechanisms and ultimately enhance yak productivity.

Recent research has shed light on the significant involvement of circular RNAs (circRNAs) in the regulation of skeletal muscle development in livestock. For example, circRILPL1 has been shown to enhance the proliferation and differentiation of myoblasts in laboratory settings, while simultaneously inhibiting apoptosis and promoting the growth of bovine myoblasts [8]. . CircMYBPC1 has been observed to engage in direct interactions with RNA-binding proteins as a competing endogenous RNA (ceRNA) in vitro. This interaction prompts the regeneration of skeletal muscle in vivo by alleviating the inhibitory effects of *MyHC* through its binding with miR-23a. This intricate mechanism plays a crucial role in the regulation of bovine skeletal muscle development, as it facilitates the downregulation of *MyHC* expression and promotes the differentiation of bovine muscle cells [9]. On the contrary, circLMO7 has been found to exert a proliferative effect and impede the differentiation process in bovine muscle cells. Conversely, miR-378a-3p has been observed to enhance the expression of the myoblast determinant *MyoD*, thereby facilitating the formation of myotubes. However, the excessive expression of circLMO7 has been associated with heightened proliferation and resistance to apoptosis in myoblasts. Furthermore, this overexpression significantly diminishes the expression of maturation markers, namely *MyoD* and *MyoG*, in bovine muscle. Additionally, circLMO7 has been shown to augment the number of myoblasts in the S phase of the cell cycle while concurrently reducing the ratio of cells in the GO/G1 phase. However, inhibiting the expression of circLMO7 can promote muscle development in cattle [10]. These multifaceted findings reveal the intricate expression patterns of circRNAs across bovine muscle tissues and underscore their diverse regulatory roles in muscle development, solidifying the ceRNA mechanism’s pivotal role in muscle growth and maturation.

The aim of this study is to investigate the expression patterns of circular RNAs (circRNAs) in the skeletal muscle of yaks under different feeding patterns. Yaks are a pivotal source of high-quality meat and milk in the Tibetan Plateau region and are recognized for their adaptability to harsh environments and limited food resources. Previous research has shown that feeding patterns have a significant impact on the growth, development, and meat quality of yaks [11, 12]. However, the molecular mechanisms underlying how feeding patterns affect yak skeletal muscle development and meat quality remain unclear. Considering that circRNAs are emerging as vital regulators of gene expression in different biological processes, including skeletal muscle development, we hypothesized that they could play a pivotal role in mediating the effects of different feeding patterns on yak muscle. Identifying individual circRNAs and their regulatory functions could provide valuable insights into the molecular mechanisms underlying yak skeletal muscle development and meat quality, thereby helping optimize feeding strategies and improve yak meat quality. To elucidate these key regulators in muscle growth and development, we constructed a ceRNA network by examining interactions among circRNAs, miRNAs, and mRNAs. These findings may contribute to improving yak breeding and providing new insights into the genetic mechanisms of muscle growth.

## Results

### Growth performance, meat quality, and carcass traits of ashdan yaks

The investigation delved into the progression and characteristics of Ashdan yak under distinct feeding conditions, namely, grazing and high-forage (HF) feeding. Results pointed towards noteworthy advancements in growth performance within the HF group, surpassing that of the grazing group (*p* < 0.01). These enhancements manifested across multiple parameters, including final body weight, average daily gain, carcass weight, slaughter rate, net meat weight, bone weight, meat-bone ratio, and eye muscle area (Refer to Table 1). Specifically, male yaks subjected to HF feeding exhibited an average body weight of 377.17 kg after a 170-day fattening period, representing a substantial 70.34 kg increase compared to the grazing group (Table 1). Similarly, the average carcass weight in the HF group surpassed that of the grazing group by 83.76 kg, underscoring the significant positive impact of fattening on the growth performance of Ashdan yak. Additionally, an evaluation of meat quality sourced from the yak’s longissimus dorsi (LD) muscle was conducted under both feeding conditions. Outcomes unveiled marked disparities (*p* < 0.01) between the two groups concerning pH at 45 min and 24 h, cooking loss, and shear force of the LD muscles. Notably, no significant distinction was observed in the muscle color index (Table 1). Taken together, these findings affirm that high-forage feeding exerts a beneficial influence on the growth performance and meat quality of Ashdan yak.

**Table 1:** Growth performance and Meat and Carcass Traits of yaks in different feeding systems

Characteristics	Group G	Group HF	*p*-value
Initial body weight (kg)	208.33 ± 12.53	215.67 ± 21.83	0.6549
Final body weight (kg)	306.83 ± 24.34B	377.17 ± 15.77 A	0.001
Average daily gain (kg)	0.52 ± 0.12B	0.86 ± 0.15 A	0.0017
Carcass weight (kg)	124.28 ± 8.77B	208.04 ± 8.75 A	< 0.001
Net meat weight (kg)	94.73 ± 4.55B	176.23 ± 10.61 A	< 0.001
Bone weight (kg)	31.08 ± 1.96b	27.17 ± 2.46a	0.012
Slaughter rate (%)	42.49 ± 10.24B	55.26 ± 14.25 A	< 0.001
Meat-bone ratio	3.46 ± 0.09B	5.68 ± 0.29 A	< 0.001
Eye muscle area (cm^2^)	57.89 ± 8.83 A	76.64 ± 10.45B	< 0.001
L* 45 min	6.32 ± 0.80	6.98 ± 1.27	0.305
a* 45 min	28.12 ± 2.32	29.29 ± 2.49	0.418
b* 45 min	5.67 ± 1.04	6.42 ± 1.38	0.311
L * 24 h	7.61 ± 0.99	8.86 ± 1.20	0.054
a* 24 h	32.68 ± 0.91	33.23 ± 1.19	0.385
b* 24 h	9.94 ± 1.18	8.62 ± 0.81	0.047
pH 45 min	6.89 ± 0.32 A	6.34 ± 0.24B	0.007
pH 24 h	5.49 ± 0.25B	6.03 ± 0.28 A	0.005
Cooking loss (%)	17.82 ± 3.84B	27.21 ± 1.70 A	< 0.001
Drip loss (%)	15.06 ± 2.30	17.27 ± 3.12	0.192
Shear force (N)	17.70 ± 1.51 A	14.69 ± 2.35B	0.0025

The average daily gain of grazing yak displays a negative value. Different lowercase letters ‘a’ and ‘b’ within the same group indicate statistical significance at p < 0.05, while uppercase letters ‘A’ and ‘B’ indicate statistical significance at *p* < 0.01 (same applies below)

### Alignment to the reference genome

In order to identify the circRNAs and mRNAs expressed during the development of the Longissimus Dorsi muscle, we constructed a total of six cDNA libraries, with three biological duplicates for each group. The libraries F2, F3, and F4 were considered replicate libraries for Group G, while Y1, Y2, and Y4 were considered replicate libraries for group HF. Overall, we obtained 665.75 million raw reads from all six libraries, a After removing low-quality reads and adapter fragments, 652 million clean reads remained, accounting for 97.93% of the total raw reads in the libraries. The Q30% values for all the libraries exceeded 93.85%, indicating high-quality clean reads. Furthermore, 94.18–95.33% of the clean reads from all the libraries were successfully mapped to the reference genome, and 90.32% of the uniquely mapped reads were used for transcript construction. (Table S1).

### Identification and characteristics of circRNA

**Fig. 1:**
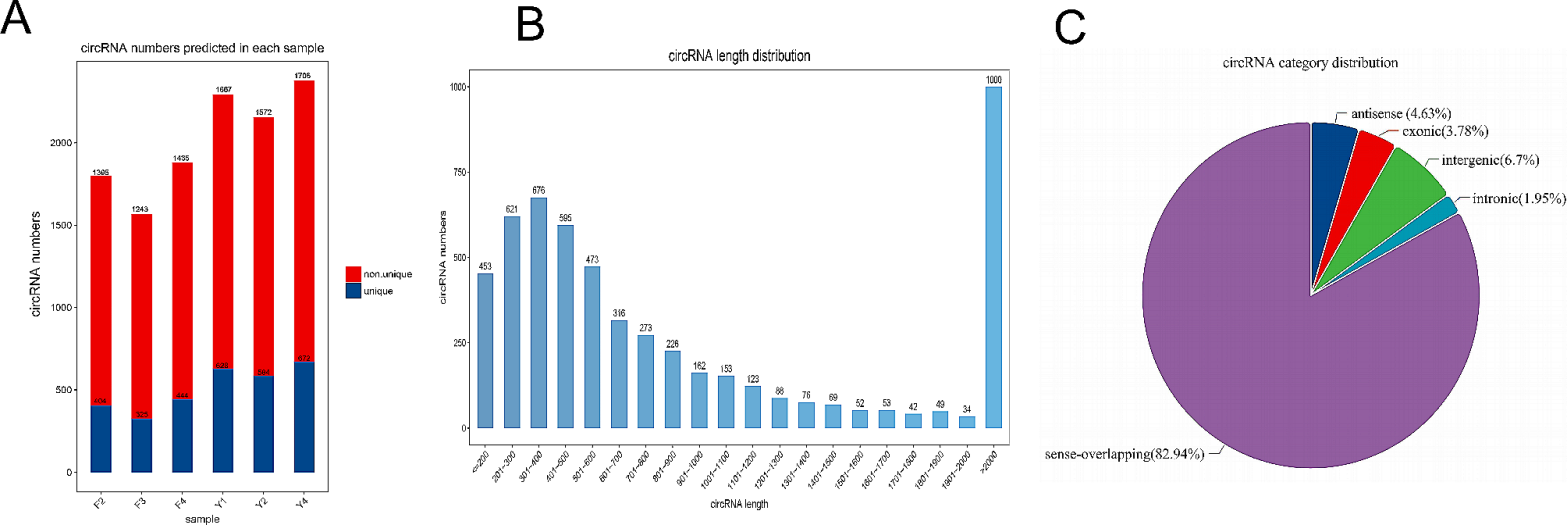
The description of identified circRNAs **(A)** circRNA numbers predicted in each sample, **(B)** The length distribution of circRNAs, **(C)** The structure type pie chart of circRNAs.

This study identified a total of 5534 circRNAs in the skeletal muscle of yaks across two feeding groups: 1749 circRNAs in group G and 2276 circRNAs in group HF (Table S2, Fig. 1A). Analyzing their expression distribution based on RBP values revealed distinct patterns among the groups, corroborating sample reproducibility and enabling further analysis (Fig. 1A). The findings of our study indicate that the average length of the identified circRNAs was 3073.44 base pairs (bp). Furthermore, it was observed that over 18.07% of the circRNAs exceeded a length of 2,000 bp (Fig. 1B). Furthermore, our analysis indicated that sense overlapping circRNAs accounted for 82.94% of the total, while only 4.63% of the antisense overlapping circRNAs were identified. The number of exonic and intronic circRNAs was 209 and 108, respectively (Fig. 1C).

### Analysis of differentially expressed circRNAs

**Fig. 2:**
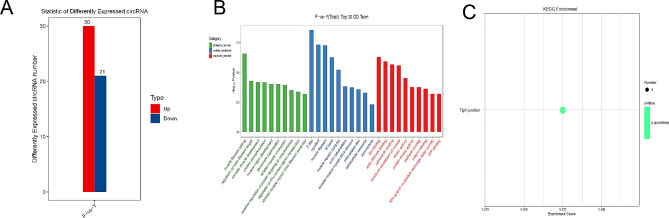
Comparative analysis of the differentially expressed circRNAs between two groups **(A)** Statistic of Differently Expressed circRNA, **(B)** GO analysis with the top 20 enrichment pathways for DEcirRNAs, **(C)** KEGG analysis with enrichment pathways for DEcirRNAs.

The primary aim of this study was to identify specific circRNAs and elucidate their regulatory functions in the development of yak skeletal muscle and meat quality. To achieve this, we employed the CIRI software to detect circRNA sequences and utilized DEGseq for evaluating differences in expression between two groups. With the implementation of a false discovery rate threshold of 0.01 and an absolute log2(fold change) value greater than 2, we were able to identify circRNAs that exhibited significant differential expression. Our analysis revealed that out of the 51 differentially expressed circRNAs between the F group and the HF group, 30 circRNAs were upregulated in the HF group while 21 circRNAs were downregulated in comparison to the F group (Fig. 2A).

The utilization of the GO and KEGG databases for functional analysis has provided valuable insights into the role of differentially expressed circRNAs in key biological processes and molecular pathways that contribute to muscle development and meat quality. Notably, the enriched GO terms encompass significant aspects such as muscle organ development (GO:0007517), myofibril assembly (GO:0030016), and muscle myosin complex (GO:0005859). Furthermore, the KEGG analysis highlights the crucial involvement of tight junction signaling (ko04530) as a pivotal pathway (Fig. 2B and C, and Table S3). These important findings shed light on the potential regulatory functions of circRNAs in determining yak meat quality, emphasizing the need for further exploration of specific circRNAs and their individual functions. Such investigations have the potential to inform the refinement of feeding strategies, ultimately leading to enhancements in yak meat quality.

### Analysis of cirRNA-miRNA-mRNA competitive regulatory network

**Fig. 3:**
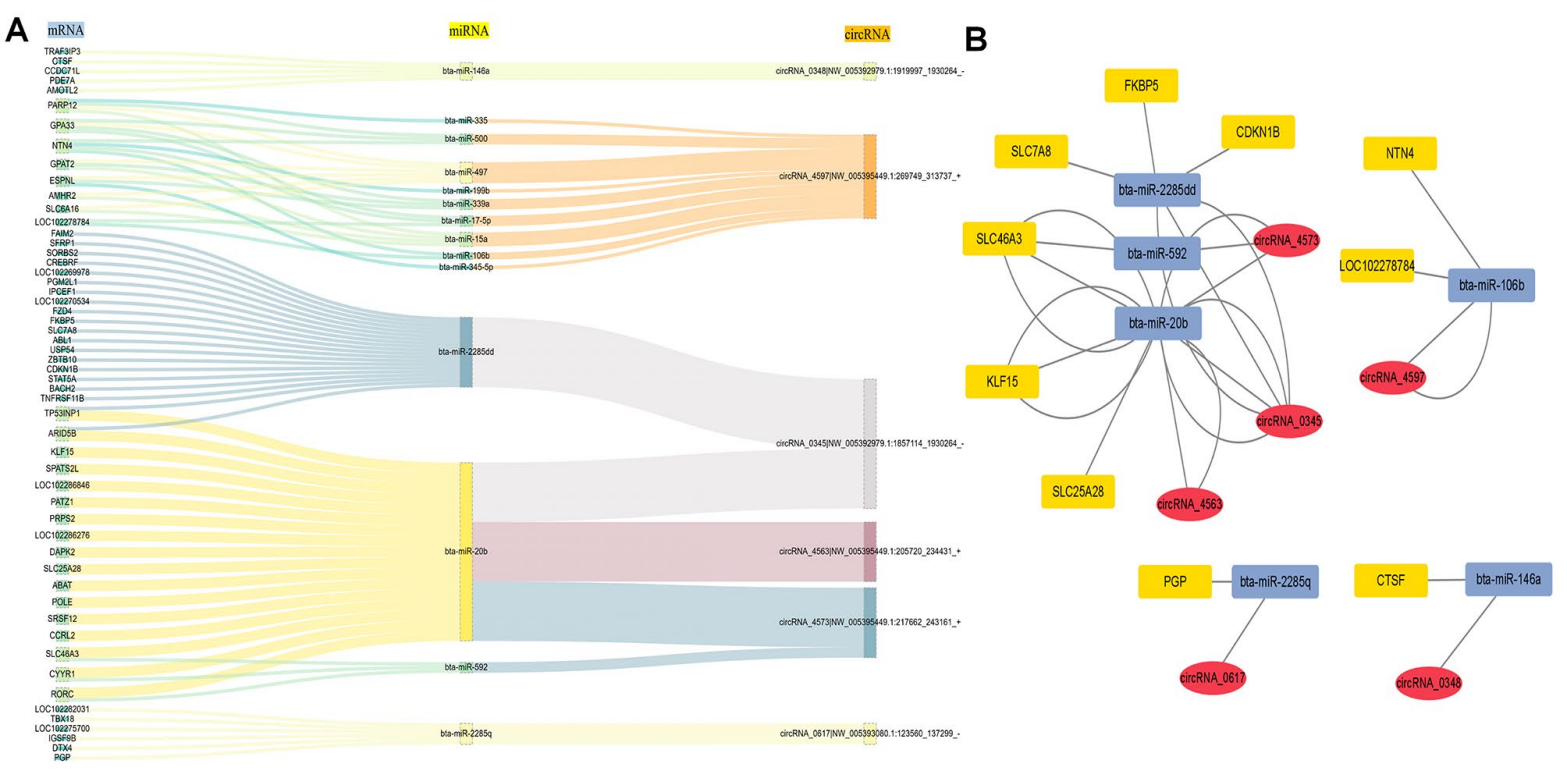
ceRNA Network in yak **(A)** Sankey diagram for the ceRNA network in yak muscle. Each rectangle represents a gene, and the connection degree of each gene is displayed based on the size of the rectangle. **(B)** The ceRNA co-regulation network, yellow boxes, blue boxes and red ovals represent differentially expressed mRNAs, circRNAs and miRNAs.

Previous studies have evidenced that mRNAs and circRNAs may regulate gene function as ceRNAs via miRNAs in various processes, thereby suggesting that ceRNAs and their corresponding miRNAs may function cooperatively [10, 13]. Consequently, an integrated ceRNA network was constructed. This network comprises 1003 DEMs, 51 DEmiRs, 52 DECs, and 33 relationships (Fig. 3A-B, Table S4). Upon studying the network, it was discerned that both *PGP* and circRNA_0617 target bta-miR-2285q. Moreover, *KLF15*, circRNA_0345, and bta-miR-20b, as well as *CTSF*, circRNA_0348, and bta-miR-146a exhibited alike results. This comprehensive ceRNA network could potentially facilitate deeper understanding of the development of longissimus dorsi and the quality of yak meat.

### Real-time quantitative PCR validation of RNA-seq

**Fig. 4:**
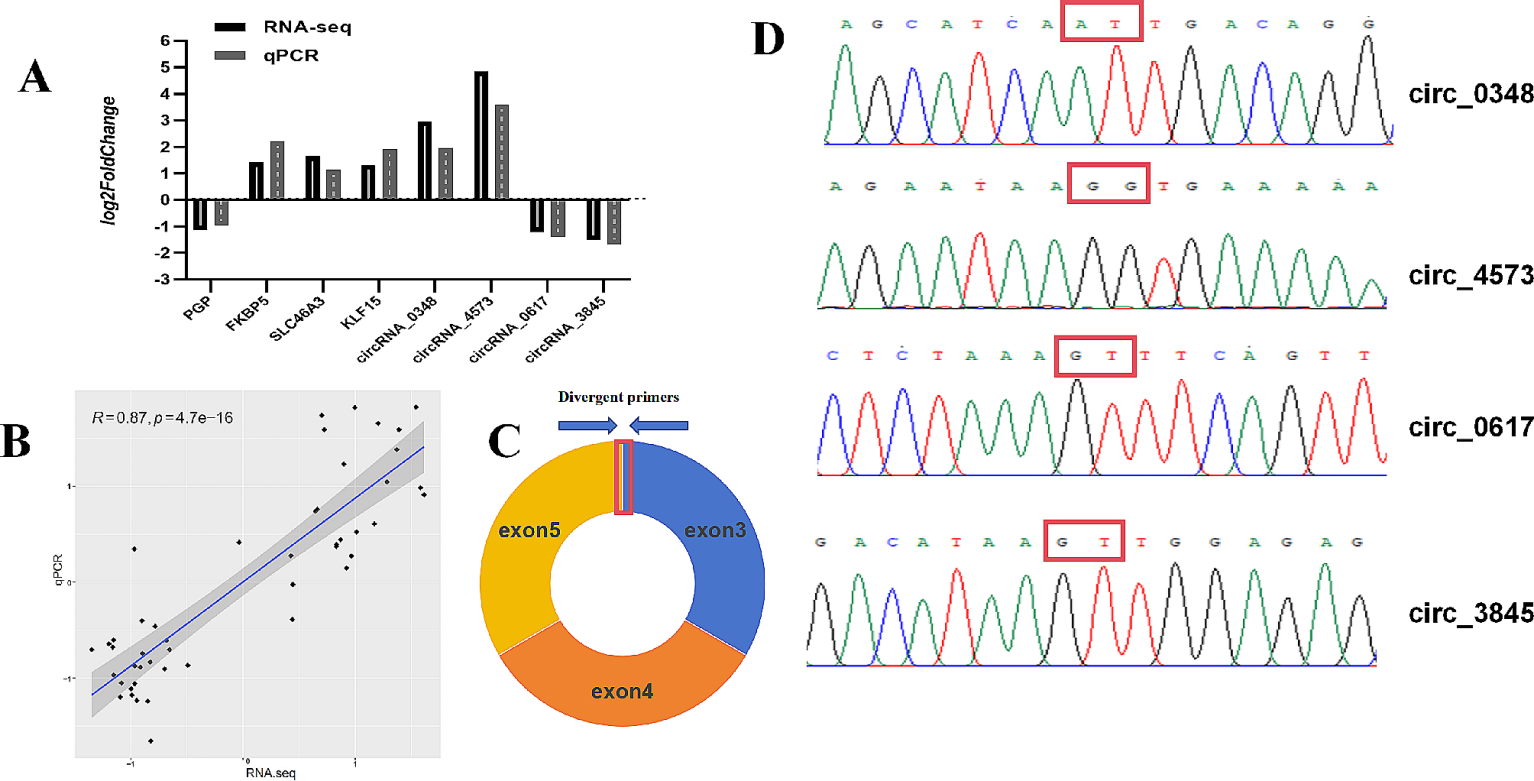
**(A)** Validation of RNA-seq by RT-qpcr analysis. **(B)** Correlation between the qRT-PCR and the RNA-seq data. The trend lines and formula in each scatter plot represent the correlation coefficients. **(C)** Divergent primers used in the amplifcation of circular junctions. **(D)** Sanger sequencing confirmed back splicing site of representative circRNA

To validate the results of the sequencing analysis, we randomly selected four circRNAs and four mRNAs for RT-qPCR analysis. To validate the authenticity of four selected circRNAs (circRNA_0348, circRNA_4573 circRNA_0617 and circRNA_3845), we employed RT-PCR amplification and Sanger sequencing. Divergent primers flanking the predicted back-splicing junction were designed for each circRNA (Fig. 4C). Successful amplification of the target sequence containing the circRNA circularization site confirmed the presence of these candidate circRNAs (Fig. 4D). The expression trends of these circRNAs/mRNAs in the tissue samples were consistent with the sequencing data, indicating that the sequencing results were reliable (Fig. 4A). Moreover, we performed a correlation analysis to assess the relationship between the expression levels of the circRNAs/mRNAs and the corresponding biological processes. This analysis provided strong evidence in support of the subsequent molecular functional validation (*R* = 0.87, *p* = 4.7e-16) (Fig. 4B).

## Materials and methods

### Weight determination in animals through feeding regimens

A group of twelve, four-year-old male Ashidan yaks were judiciously selected from the Datong Yak Farm in Qinghai Province, and consequently divided into two distinct groups: Group G (grazing) and Group HF (in-house feeding), each comprising six yaks. This experiment was coordinated on the same farm from April 5th to October 2nd, 2019. The yaks in Group G were allotted unrestricted grazing access within the farm’s pasture, whereas those in Group HF received their feed indoors. The experimental phase spanned 170 days, following an initial pre-trial tenure of 10 days. The diet administered encompassed both concentrate, made from commonly utilized feed materials, and coarse feed that included oat hay and alfalfa hay. An exhaustive elaboration on the composition, as well as nutrient levels of the experimental diet, is outlined in a previous work [14]. Prior to the feeding process, all yaks were subjected to a deinsectization treatment. Yaks in Group HF were fed in two shifts - morning and evening, with unrestricted access to drinking water throughout. The consumption levels of the concentrate were meticulously documented.

### Tissue collection

Upon completion of the feeding trial, all yaks entered a period of water deprivation from 8:30 a.m. to 10:30 a.m. Prior to sample collection, each yak received a hip injection of LuMianNing (Xylazine Hydrochloride Injection, lot number 070011777, manufactured by Jilin Huamu Animal Health Products Co., Ltd, Jilin, China) with a dosage of 0.002 mg/kg, to induce deep anesthesia. The anesthetized yaks were then euthanized via arterial bleed, carried out professionally in a slaughterhouse. Following a 24-hour fasting period, the yaks were slaughtered; both their carcass and live weights were recorded along with measurements of the eye muscle area and slaughter rate [15]. After slaughter, the pH value, meat color, shear force, cooking loss, and drop loss rate of the 12th to 13th intercostal longissimus dorsi (LD) muscle were evaluated at 45 min and again at 24 h post-slaughter [12]. Furthermore, from each experimental group, six LD muscle samples were randomly selected, portioned into small pieces (1–3 cm^3^), and stored in cryo-storage tubes. These samples were transported in dry ice to the laboratory for long-term storage at -80 °C.

### Library preparation and RNA‑seq

Total RNA was isolated from the LD tissue via the TRIzol reagent (Invitrogen, Carlsbad, CA, USA), and subsequent quality assessment ensued. The integrity of the RNA underwent scrutiny through 1% agarose gel electrophoresis, while the Agilent 2100 assay, RIN, and analysis of 28 S/18 or 23 S/16S were employed to determine concentration and purity. In the case of standard animals, the TruSeq Stranded Total RNA with ribo-zero gold kit (Illumina, San Diego, CA, USA) was applied. DNase I (Fermentas, Vilnius, Lithuania) was used to eradicate genomic DNA from the samples, and cDNA synthesis from 2 µg of total RNA in nine samples was conducted using the Illumina HiSeqTM 2500 system (Illumina Corp, San Diego, CA, USA), adhering to the manufacturer’s guidelines. Quantitative analysis was performed utilizing an Agilent 2100 Bioanalyzer (Agilent, Santa Clara, CA, USA), and specific strand libraries underwent sequencing on an Illumina HiSeqTM 2500 instrument, generating 150-nt paired-end sequences. Library construction and sequencing were executed using the Illumina platform by OE Biotech Co. (Shanghai, China). The raw Illumina sequencing data underwent quality assessment with FASTQC tools [16]. Trimmomatic with sliding windows was utilized to reduce and trim the quality of each substrate at the 3’-end, resulting in an average base quality of 90% accuracy [17]. The trimmed reads were also rechecked for quality using the FASTQC tool. HISAT2 [18] was employed to align the remaining high-quality clean reads with the yak genome (BosGru_v2.0). The mapped read segments for each sample were assembled using StringTie (v1.3.1) [19] facilitated the assembly of mapped read segments for each sample through a reference-based approach, and the ensuing transcript underwent annotation using the Cuffcompare program within the Cufflinks package.

### Identification of circular RNAs in yak muscle

In the circRNA expression analysis, the CIRI software program was utilized [20]. Initially, CIRI was employed to detect paired chiastic clipping signals (PCC) and predict circRNA sequences based on junction reads and GT-AG cleavage signals. Subsequently, the RPM algorithm (Reads Per Kilobase per Million reads) was used to quantify the expression levels of circRNAs.

To evaluate the differential expression of circRNAs between two groups, the DEGseq software [21] was applied, with a false discovery rate (FDR) threshold of 0.01. Significantly differentially expressed circRNAs were identified based on a log2(fold change) absolute value greater than 2. The GO and KEGG databases were then utilized for further analysis. Validation of the results was conducted using the Hypergeometric Distribution Test, and the Benjamini and Hochberg method [22]. was employed for multiple test adjustments. Enrichment terms with a p-value below 0.05 were considered statistically significant. The DAVID database was used to map the host genes of differentially expressed circRNAs to GO terms and KEGG pathways in the GO and KEGG analysis. Subsequently, the enriched GO terms and KEGG pathways were analyzed to identify potential biological functions and pathways associated with the circRNAs.

### Construction of ceRNA Network

To enhance our understanding of the reciprocity among mRNAs, circRNAs, and miRNAs, we crafted a circRNA–miRNA–mRNA regulatory network, underpinned by the ceRNA hypothesis [23]. This proposition suggests circRNAs, mRNAs, and miRNAs interplay, governing each other’s roles as competing endogenous RNAs (ceRNAs). Utilizing MiRanda [24] ,we predicted the potential binding pairs for miRNA–mRNA and miRNA–circRNA respectively. Further, the correlation intensity between each miRNA–mRNA pair and miRNA–circRNA pair was examined using the Spearman Correlation Coefficient (SCC). Pairs showcasing an SCC value exceeding 0.8 were deemed significant for network construction, with the statistical significance threshold set at *p* < 0.05. The resulting network was visually represented using the Cytoscape software (version 3.5.1).

### Real-time quantitative PCR validation of sequencing data

In this study, we investigated the expression of RNA in yak LD tissue across different groups. We used RT-qPCR to measure the expression levels of eight DEGs and DECs. The genes analyzed were *PGP, FKBP5, SLC46A3, KLF15*, circRNA_0348, circRNA_4573, circRNA_0617, and circRNA_3845. The primer sequences required for amplifying these genes can be found in Table S5. β-actin was used as the internal reference gene for mRNA expression, while 18 S served as the internal reference for circRNAs. The qRT-PCR was performed on the LightCycler® 480 II Real-time PCR Instrument using a 10-µL PCR reaction mixture. The reactions were carried out in a 96-well optical plate with specific thermal cycling conditions. The expression levels of mRNA and circRNAs were determined using the 2-ΔΔCt method, with β-actin and 18 S as the reference genes, respectively.

## Discussion

Yaks are raised in harsh environments throughout the year, including a dry season. However, overpopulation of yaks has led to grassland degradation and decreased growth performance [25]. Rearing yaks indoors can enhance their productive performance and yield economic benefits. This was corroborated by our experiment, where the high feed (HF) group manifested a superior average daily gain and net body weight compared to the grazing (G) group. Yak meat tenderness, a significant factor affecting meat quality and consumption, is often compromised by extended grazing periods and older slaughtering ages, thereby restricting its market appeal. Studies suggest that indoor rearing can augment the intramuscular fat (IMF) content in the Longissimus dorsi (LD) muscle, consequently diminishing shear stress and enhancing meat tenderness [6, 26]. The pH value of muscles also affects meat quality, with lower pH values leading to tougher and less colorful meat. Muscle glycogen is converted to lactic acid post-slaughter, which lowers pH values and can affect meat tenderness and color by reducing proteolysis [27]. Our study reveals that proper feeding can enhance the tenderness of yak meat, as the HF group exhibited significantly lower shear force and a slower rate of pH value decrease compared to the G group.

Skeletal muscle, a crucial organ in animals, performs pivotal roles in motor functions and serves as an integral endocrine and metabolic organ correlated with growth and development [28, 29]. Its structure comprises myofibers, formed from the fusion of myogenic cells into multinucleated myotubes, further aggregating with connective tissue and capillaries to create myofascicles [30]. Recent years have witnessed growing interest in circRNAs, non-coding RNAs characterized by unique expression patterns and marked stability within organisms. Current studies increasingly explore their biological functions in organism development and tumorigenesis. Existing evidence indicates circRNAs’ significant roles in muscle development in sheep [31], cattle [32], goat [33], and monkey [34]. However, the identification and characterization of circRNAs in yak, especially those associated with skeletal muscle development, largely remain elusive. This study aims to identify and characterize circRNAs within the yak’s LD muscle for the first time, successfully identifying 5534 circRNAs across two groups of skeletal muscle.

Recent discoveries continue to emphasize the significant role of functional circRNAs, as a transcriptional product, in various tissues and cell types in animals [8, 10, 13]. We identified 51 differentially expressed circRNAs; 20 were up-regulated and 31 were down-regulated across two distinct groups. These circRNAs are likely associated with potential biological functions related to yak muscle development, myoblast proliferation and differentiation, which include muscle organ development (GO:0007517) and Tight Junction pathway (ko04530). Tight junctions (TJs), a type of intercellular junction, play a fundamental role in maintaining tissue barrier functions, including in skeletal muscle [35]. They exist in the capillary endothelial cells and pericytes of skeletal muscle and are crucial for regulating the exchange of nutrients and waste products between muscle cells and blood vessels [36]. Nonetheless, the role of tight junction pathways in skeletal muscle development under varying feeding conditions remains to be explored. Important to mention are genes associated with circRNAs in the TJs pathway, such as circRNA_4563-MYH1 and circRNA_4573-*MYH1*, imperative for *MYH2* expression. In the HF group, gene 9 (*MYH2*) exhibited higher expression than in the G group (log2FoldChange = -0.88, *p* = 0.096), whereas the expression of *MYH1*, *MYH7*, and *MYH4* did not significantly differ (Table S4). The differential expression of *MYH2* was significantly higher, implying an influence of glycolysis on the conversion of Type IIB muscle fibers to IIA. Previous research indicates an increase in Type IIB muscle fibers corresponds to a decrease in muscle pH and meat tenderness, while an increase in Type IIA fibers enhances muscle tenderness [37]. This was corroborated by our study; the HF group showed lower shear force and higher pH at 24 h compared to the G group.

Advances in sequencing technology have illuminated the regulatory roles of non-coding RNAs (ncRNAs) in myogenesis [38, 39]. Among them, Circular RNAs (circRNAs) stand out due to their unique covalently closed loop structure, contrasting with the relatively short length of microRNAs (miRNAs) which are approximately 22 nucleotides long. While the functions of circRNAs remain largely uncharted, they are known to act as miRNA sponges, impacting mRNA expression in the process [40]. In our research, we developed an interaction network involving mRNA, circRNAs, and miRNAs, and found that several circRNAs regulate the expression of genes vital for the development of yak skeletal muscle and the traits influencing meat quality. Moreover, we identified target genes of miRNAs within the constructed mRNA-circRNA-miRNA network, providing insights into the intricate regulatory mechanisms that govern biological processes. Our research revealed that *PGP* and circRNA_0617 target bta-miR-2285q. *PGP* (phosphoglycolate phosphatase), a pivotal component in skeletal muscle development, significantly influences meat quality. Previous research indicates PGP’s role in the glycolytic pathway, which is essential for energy production in skeletal muscle. These studies also suggest that phosphoglycolate phosphatase could affect the changes in the beef proteome related to tenderness, by potentially regulating muscle metabolism and oxidative stress, both of which are key determinants of meat tenderness [41, 42]. In the HF group, we noted a considerable reduction in tenderness in comparison to the F group, a finding consistent with previous research. Nonetheless, further rigorous experimentation is requisite to develop a more comprehensive understanding of the link between phosphoglycolic acid phosphatase and yak meat tenderness. These interaction networks, integral to myogenesis, herald fresh perspectives in muscle formation research and constitute a stride towards enhancing meat quality.

## Conclusion

Our study emphasizes the pivotal role indoor feeding practices play in augmenting yak performance and meat quality. The identification and subsequent comprehensive characterization of circRNAs in yak skeletal muscle have facilitated deeper insights into the regulatory mechanisms tied to yak skeletal muscle development and traits affecting meat quality. As such, our findings lay the groundwork for future investigations in this field. The ceRNAs network we established further underscores the critical impact of non-coding RNAs on yak skeletal muscle development and traits related to meat quality. These observations contribute novel insights into the circRNAs’ regulatory functions within yak skeletal muscle development and meat quality, with potential applications in the management of yak populations and the production of prime quality yak meat.

### Electronic supplementary material

Below is the link to the electronic supplementary material.

Supplementary Material 1: Table S1: Summary of RNA-seq data and reads mapped to the yak reference genome.

Supplementary Material 2: Table S2: circRNAs detected at each group.

Supplementary Material 3: Table S3: Total enriched KEGG pathways and GO terms of DECs.

Supplementary Material 4: Table S4: The summary of DEMs, DECs and DEmirs of yaks.

Supplementary Material 5: Table S5: Primer pairs used for Real-Time Quantitative amplification.

## Data Availability

The datasets generated and/or analysed during the current study are available in the NCBI repository, PRJNA893108.

## Abbreviations

DEGs: Differentially expressed genes
GO: Gene ontology
KEEG: Kyoto encyclopedia of genes and genomes
Group G: Grazing group
Group HF: In-house feeding
FDR: False discovery rate
FC: Fold change
LD: Longissimus dorsi muscle
qRT-PCR: Quantitative real-time PCR
TJs: Tight junctions
DEGs: Differentially expressed genes
DECs: Differentially expressed circRNAs
DEmiRs: Differentially expressed miRNAs
